# Decreased expression of zinc-alpha2-glycoprotein in hepatocellular carcinoma associates with poor prognosis

**DOI:** 10.1186/1479-5876-10-106

**Published:** 2012-05-24

**Authors:** Yan Huang, Lin-Zi Li, Chris Zhi-Yi Zhang, Chun Yi, Li-Li Liu, Xuan Zhou, Guo-Bing Xie, Mu-Yan Cai, Yan Li, Jing-Ping Yun

**Affiliations:** 1 Department of Pathology, State Key Laboratory of Oncology in South China, Sun Yat-Sen University Cancer Center, Guangzhou, China, Sun Yat-Sen University Cancer Center, No 651, East Dongfeng Road, Guangzhou, 510060, China

**Keywords:** AZGP1, Hepatocellular carcinoma, Prognosis, Recurrence, Metastasis

## Abstract

**Background:**

Zinc-alpha2-glycoprotein (AZGP1, ZAG) was recently demonstrated to be an important factor in tumor carcinogenesis. However, AZGP1 expression in hepatocellular carcinoma (HCC) and its significance remain largely unknown.

**Methods:**

Quantitative real-time polymerase chain reaction (qRT-PCR) was applied to determine mRNA level of AZGP1 in 20 paired fresh HCC tissues. Clinical and pathological data of 246 HCC patients were collected. Tissue-microarray-based immunohistochemistry (IHC) was performed to examine AZGP1 expression in HCC samples. Relationship between AZGP1 expression and clinicopathological features was analyzed by Chi-square test, Kaplan-Meier analysis and Cox proportional hazards regression model.

**Results:**

AZGP1 expression was significantly lower in 80.0% (16/20) of tumorous tissues than that in the corresponding adjacent nontumorous liver tissues (*P* < 0.001). Consistently, IHC data revealed that decreased expression of AZGP1 was present in 80.1% (197/246) of HCC patient tissues (*P* < 0.001). Furthermore, AZGP1 expression in HCC significantly associated with several clinicopathological parameters, including serum AFP level (*P* = 0.013), liver cirrhosis (*P* = 0.002) and tumor differentiation (*P* = 0.025). Moreover, HCC patients with high AZGP1 expression survived longer, with better overall survival (*P* = 0.006) and disease-free survival (*P* = 0.025). In addition, low AZGP1 expression associated with worse relapse-free survival (*P* = 0.046) and distant metastatic progression-free survival (*P* = 0.036).

**Conclusion:**

AZGP1 was downregulated in HCC and could be served as a promising prognostic marker for HCC patients.

## Background

Hepatocellular carcinoma (HCC), one of the most popular tumors in the whole world, is the third leading cause of tumor mortality [1]. The incidence of HCC varies greatly around the world, with the highest prevalence in Southeast Asia and Africa. About 230,000 people (53% of the world cases) in China die from HCC every year [2]. Hepatocarcinogenesis is a complex process and the occurrence is the result of coactions of multi-factors, such as physical condition, aflatoxin, hepatitis virus infection, cirrhosis, genetic susceptibility and epigenetic changes [3,4]. The main effective treatments of HCC include surgery, radiofrequency, combination of chemotherapeutics and radiotherapy recently, whereas post-treatment relapse and metastasis are dangerous factors of therapeutic effect [5,6]. Although the development of multidisciplinary treatment brings us so many advantages and disadvantages, it is urgent and necessary to identify specific markers which could predict recurrence, metastasis and prognosis for patients with HCC to improve the individual treatment. Some crucial genes are been raised to assist tumor treatment, such as BRAF and PIK3CA [7].

Zinc-alpha2-glycoprotein (AZGP1), a 40-kDa single-chain polypeptide assigned to the chromosome 7q22.1, is involved in carcinogenesis and differentiation. The structure and sequence of AZGP1 are highly homology to major histocompatibility complex class I (MHC I) family which may function importantly in immunity [8]. AZGP1 is a member of macroglobulin family, which is actively involved in both inhibition of tumor growth and proliferation [9]. AZGP1 was reported to inhibit the enzyme-mediated tumor invasion and activate apoptosis though binding hydrolases [10]. AZGP1 normally distributed in human liver, prostate, breast,esophagus and other organs [11]. AZGP1 has been reported to inhibit the proliferation of tumor cells by down-regulating cdc2 so as to lead to a cell cycle arrest in G2/M phase [12]. Besides, the capacity of AZGP1 to inhibit TGF-β-mediated epithelial-to-mesenchymal transdifferentiation (EMT) makes it possible to inhibit tumor invasion [13]. Apart from the above-mentioned function, there is mounting evidence to show AZGP1 may play a part in the expression of the immune response [8]. As the structure and sequence are highly homology to MHC class I, AZGP1 may afford some protective effect in tumor patients and benefit to prevent the cancer progression [14].

Recent studies indicated that AZGP1 was differently expressed in human tumors. For example, its expression is increased in breast cancer, prostate cancer and lung adenocarcinoma [14-16], but decreased in pancreatic carcinoma and oral tumors [13,17]. Interestingly, expressions of AZGP1 in cancer tissues have been demonstrated to be connected with recurrence and metastasis [18,19].

However, the expression of AZGP1 in HCC and its significance is still unknown. In this study, we examined the mRNA and protein levels of AZGP1 in HCC and determined the relationship between AZGP1 expression and various clinicopathologic parameters in order to systematically investigate whether AZGP1 plays a role in HCC carcinogenesis and its clinical significance.

## Materials and methods

### Patients and tissue specimens

Paraffin-embedded specimens from 246 patients with HCC between 1997 and 2001 by immunohistochemistry, as well as clinical and pathological data, and 20 cases of resected fresh tissues with HCC between Jan and Jul 2011 for analysis of mRNA and protein expression, were collected from Sun Yat-Sen University Cancer Center, Guangzhou, China. The 246 cases, with histologically conformed were randomly selected from 400 patients with HCC who underwent initial surgical resection and did not receive preoperative radiotherapy or chemotherapy. Child-Pugh classification and the 15-min retention rate for indocyanine green (ICGR15) were essential to be considered in the preoperative assessment. The cases selected were based on availability of 10 years’ follow-up data and complete medical records. Patients whose cause of death remain unknown were excluded form our study. The 246 cases aged from 14 to 78 years (mean, 48 years) include 219 males (91.3%) and 27 females (8.7%). Clinicopathologic characteristics for these patients were described in Table 1. Tumor differentiation was assessed according to the criteria proposed by Edmonson and Steiner [20]. Tumor stage was defined according to American Joint Committee on Cancer/International Union Against Cancer tumor-node-metastasis (TNM) classification system [21]. The use of tissues for this study was approved by the Institute Research Medical Ethics Committee of Sun Yat-Sen University Cancer Center and written informed consent was obtained from each patient.

**Table 1:** Correlation between AZGP1 expression and clinical and pathological characters in HCC

**Variable**	**AZGP1 protein**				
	**All cases**	**Low expression**	**High expression**	** *χ* 2**	**P value**^**α**^
Age(years) b				0.134	0.714
48	119	46 (38.7)	73 (61.3)		
≥ 48	127	52 (40.9)	75 (59.1)		
Mean ± SD(47.9 ± 11.8)					
Gender				0.099	0.753
Female	27	10 (37.0)	17(63.0)		
Male	219	88(40.2)	131(59.8)		
HBsAg				0.673	0.412
Yes	213	87 (40.8)	126 (59.2)		
No	33	11(33.3)	22 (66.7)		
AFP (ng/ml)				6.182	**0.013**
≤ 20	104	32 (30.8)	72 (69.2)		
> 20	142	66 (46.5)	76 (53.5)		
Liver cirrhosis				9.244	**0.002**
Yes	177	81 (45.8)	96 (54.2)		
No	69	17 (24.6)	52 (75.4)		
Tumor size (cm)				0.457	0.499
≤ 5	127	48 (37.8)	79 (62.2)		
> 5	119	50 (42.0)	69 (58.0)		
Tumor multiplicity				0.042	0.837
Single	130	51 (39.2)	79 (60.8)		
Multiple	116	47 (40.5)	69 (59.5)		
Tumor differentiation				9.347	**0.025**
Well (I)	33	6 (18.2)	27 (81.8)		
Moderate (II)	125	54 (43.2)	71 (56.8)		
Poor (III)	76	35 (46.1)	41 (53.9)		
Undifferentiation (IV)	12	3 (25)	9 (75)		
Clinical Stage				2.154	0.541
I	16	5 (31.3)	11 (68.7)		
II	93	33 (35.5)	60 (61.5)		
III	106	46 (43.4)	60 (56.6)		
IV	31	14 (45.2)	17 (54.8)		
Vascular invasion				1.966	0.161
Yes	73	34 (46.6)	39 (53.4)		
No	173	64 (37.0)	109 (63.0)		

^α^Chi-square test; b patients were divided according to the median age; *AFP*, alpha-fetoprotein; *HBsAg*, hepatitis *B* surface antigen; *AZGP1*, zinc-alpha2-glycoprotein.

### RNA preparation and quantitative real-time polymerase chain reaction

Total RNA was extracted from 20 pairs of HCC tissues and the adjacent non-tumorous liver tissues from 20 cases of fresh tissues with HCC using Trizol regent (BIOO Scientific Corp., Austin, TX,USA) and cDNA was synthesised using SuperScript RT kit (Promega, Madison, WI, USA) according to the manufacturer's instructions. The expression levels of AZGP1 and β-actin were measured using SYBR green-based real-time PCR performed on the Stratagene Mx3000P Real-Time PCR system. The primer sequences for AZGP1 were sense, ACGACAGTAACGGGTCTCAC and antisense, AGGCTGGGATTTCTTTGTTGAA, whereas those for β-actin were sense, TGGCACCCAGCACAATGAA and antisense CTAAGTCATAGTCCGCCTAGAAGCA. The optimised amplification protocol consisted of an initial denaturation step of 95°C for 10 min, followed by 40 amplification cycles at 95°C for 10 s, annealing at 59°C for 20 s and elongation at 72°C for 10 s. The fold-changes for AZGP1 expression levels were calculated using 2-ΔΔCt.

### Western blot

The 20 pairs of fresh samples including tumor tissues and the adjacent non-tumorous liver tissues were homogenated in a RIPA lysis buffer, and lysates were cleared by centrifugation (12,000 rpm) at 4°C for 30 min. The supernatant was collected and total protein (50 μg) was separated on 12% SDS-polyacrylamide gel electrophoresis (PAGE), and electrotransferred on a polyvinylidene difluoride (PVDF) membrane (Pall Corp., Port Washington, NY). After blocking non-specific binding sites for 1 h with 5% non-fat milk, the membranes were then incubated with primary mouse monoclonal antibodies against AZGP1 (Santa Cruz Biotechonlogy, CA, USA, at 1:1000 dilution), and GAPDH (Santa Cruz Biotechonlogy, CA, USA, at 1:4000 dilution) at 4°C overnight. After washing, the membrane was then incubated with the secondary anti-mouse antibody (Santa Cruz Biotechnology, CA, USA) for 1 h at room temperature. The immunoreactive signals were detected with enhanced chemiluminescence kit (Amersham Biosciences, Uppsala, Sweden). The procedures followed were conducted in accordance with the manufacturer’s instructions.

### Tissue microarray (TMA)

Tissue microarray was constructed according to the method described previously [22]. Briefly, all samples were fixed in formalin and embedded in paraffin. The corresponding histological hematoxylin and eosin (H&E)-stained sections were reviewed by two pathologists to determine and mark out representative areas of the paraffin blocks. Each case had four core biopsies, which contained two tumor tissues and two adjacent non-tumorous liver tissues. Each tissue core with a diameter of 0.6 mm was punched from the marked areas and re-embedded into a recipient paraffin arrayblock. Multiple sections (3-μm thick) were cut and mounted on microscope slides. After then, one section from the tissue array block was stained with H&E and confirmed that the punches had got the representative areas.

### Immunohistochemistry

Immunohistochemical detection for AZGP1 was performed using a standard two-step method. The TMA sections were dried overnight at 37°C, deparaffinized in xylene and hydrated through a series of graded alcohol. Before antigen retrieval, endogenous peroxidase activity was inhibited with 3% hydrogen peroxide for 20 min. Then the slides were boiled in ethylene diamine tetraacetic acid (EDTA; 1 mmol/L; PH 8.0) by a pressure cooker for 5 min. After washing five times in phosphate buffered saline (PBS; 0.01 mol/L; pH = 7.4), the slides were preincubated with 10% normal goat serum for 30 min to reduce nonspecific antibody binding. Subsequently, they were incubated overnight at 4°C with the rabbit polyclonal antibody against AZGP1 (Protein Tech Group, Chicago, IL, USA; 1:800 dilution). After rinsing, the slides were incubated with a secondary antibody (Dako Corp., Glostrup, Denmark) for 1 h at room temperature, and stained with 3, 3-diaminobenzidine tetrahydrochloride (DAB) after washing in PBS again. Finally, they were counterstained with Mayer's hematoxylin, dehydrated, and mounted. Slides were immunoreacted with PBS as the negative controls. Known immunostaining-positive slides were used as positive controls.

### Immunohistochemistry evaluation

Semi-quantitative immunohistochemical detection was used to determine the AZGP1 protein levels. Cytoplasm immunoreactivity for the AZGP1 protein was scored by evaluating the sum of positive tumor cells and the staining intensity over the total number of tumor cells. In brief, the percent of positive cells was scored as "0" (0%), "1" (1%-10%), "2" (11%-50%), "3" (51%-80%), "4" (81%-100%). Intensity was scored as "0" (no staining), "1" (weakly stained), "2" (moderately stained), and "3" (strongly stained). Both the scores were decided under double-blind conditions by three independent professionals. The final immunoreactive scores were determined by multiplying the intensity scores with the extent of positivity scores of stained cells [23,24]. The scores were then classified as follow: "-" (score 0), " + " (score 1–4), "++" (score 5–8), and "+++"" (score 9-12). For the purpose of statistical analysis, the cohort was grouped into low expression group ("-"," + ") and high expression group ("++","+++") for either 246 tumor tissues or their adjacent non-tumorous liver tissues.

### Statistical analysis

Statistical analyses were performed with the SPSS 13.0 software (SPSS, Chicago, IL, USA). The correlation between AZGP1 expression and various clinical and pathological characteristics were evaluated by Paired sample *t*-test. Kaplan-Meier analyses were used to analyze AZGP1 as univariate in prediction of patients’ survival. Comparisons of survival distributions were done with log-rank test. The multivariate survival analyses were performed by a stepwise Cox proportional hazard model using the Wald statistics. *P* values of < 0.05 were considered significant.

## Results

### The expression of AZGP1 in HCC by qRT-PCR and western blotting

**Figure 1:**
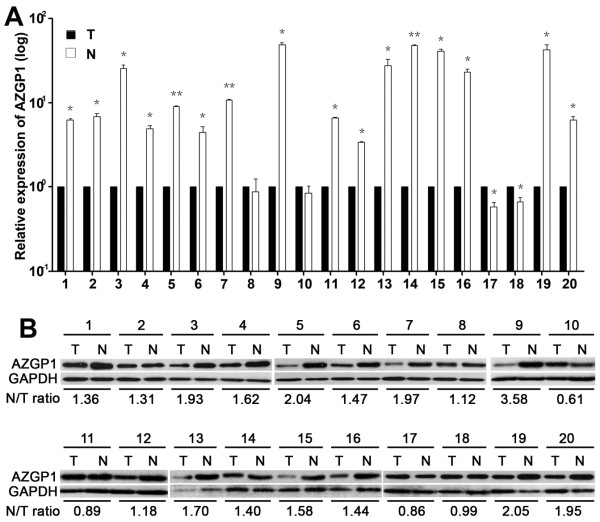
**The expression of AZGP1 in HCC by qRT-PCR and Western blotting. A.** Expression of AZGP1 mRNA in HCC tissues (T) and the adjacent non-tumorous liver tissues (N) (n = 20) was examined by qRT-PCR. Data were mean ± SD of three independent experiments. *, *P* < 0.05 and **, *P* < 0.001 versus to the adjacent non-tumorous liver tissues. **B.** Expression of AZGP1 protein in HCC tissues (T) and the adjacent non-tumorous liver tissues (N) (n = 20) was examined by Westen blotting. Ratio of AZGP1 expression in N to T was indicated.

Results showed that in 16 out of 20 cases, AZGP1 mRNA expressions in HCC tissues were noticeably lower than those in the adjacent non-tumorous liver tissues, as determined by qRT-PCR. As depicted in Figure 1A, mRNA level of AZGP1 in HCC tissue was on average 15.5-fold lower than that in corresponding adjacent liver tissue. There was significant difference in the average level of AZGP1 between tumor tissues and non-tumorous tissues (*P* = 0.001). To examine the protein level of AZGP1 in HCC, western blot was performed (Figure 1B). The ratio of AZGP1 expression in non-tumorous tissues to that in tumorous tissues was calculated. Results showed that AZGP1 expression in tumorous tissues was on average 1.55-fold lower than that in the paired non-tumorous tissues.

### The expression of AZGP1 in HCC by immunohistochemistry

**Figure 2:**
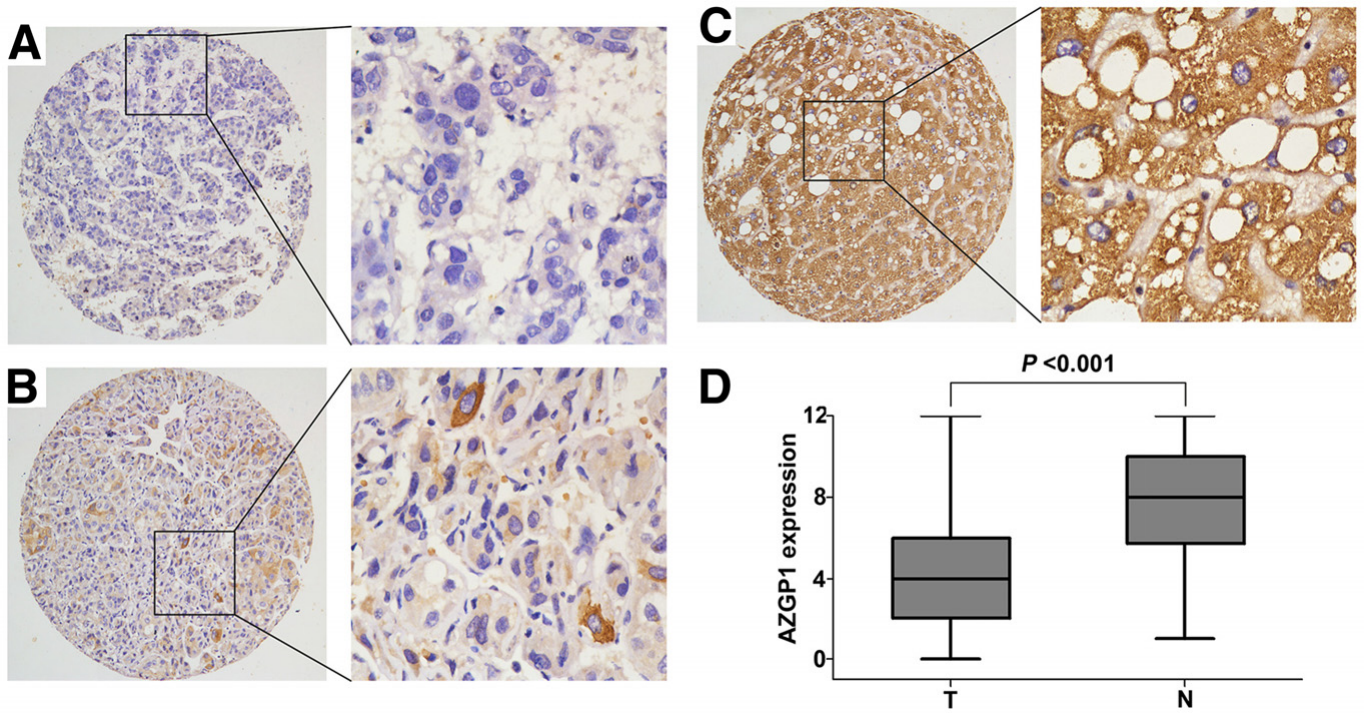
**The protein expression of AZGP1 in HCC by immunohistochemistry.** The immunoreactivity was primarily observed in the cytoplasm within tumor cells. **A.** Negative staining of AZGP1 was detected in HCC case (#136) **B.** HCC case (#57) showed weak expression of AZGP1. **C.** Strong staining of AZGP1 was observed in 95% of the adjacent non-tumorous liver tissues. **D.** The box plot showed the mean staining score of AZGP1 in HCC tissues (T) and the adjacent non-tumorous liver tissues (N) (*P* < 0.001, *t* = -6.502).

The protein status of AZGP1 was further examined by TMA and IHC, using 246 pairs of HCC and adjacent non-tumorous liver tissues (Figure 2). Results indicated that reduced expression of AZGP1 presented in 80.1% of HCC tissues (Figure 2A; C). Kruskal-Wallis's analysis revealed that AZGP1 expression was significantly decreased by more than 2-fold in tumorous tissues, compared to that in adjacent non-tumorous liver tissues (*P* <0.001, *t* = -6.502) (Figure 2D).

### The relationship between AZGP1 expression and clinicopathological parameters

To investigate the relationship between AZGP1 expression and clinicopathological parameters in 246 cases with HCC, these cases were first divided into two subgroups: “Low AZGP1 expression” and “High AZGP1 expression” as defined in the Immunohistochemistry section of Materials and Methods. Significant correlations were found between AZGP1 expression and three parameters including serum AFP levels (*P* = 0.013), cirrhosis (*P* = 0.002) and tumor differentiation (*P* = 0.017). Patients with low AZGP1 expression had a higher tendency to have high level of serum AFP, liver cirrhosis, and poor tumor differentiation. There were no statistical connections between AZGP1 expression and the rest clinicopathological parameters, such as patient age, gender, HBsAg, tumor size, tumor multiplicity, clinical stage and vascular invasion (*P* > 0.05, Table 1).

### The association of low AZGP1 expression in HCC with poor survival in patient

**Figure 3:**
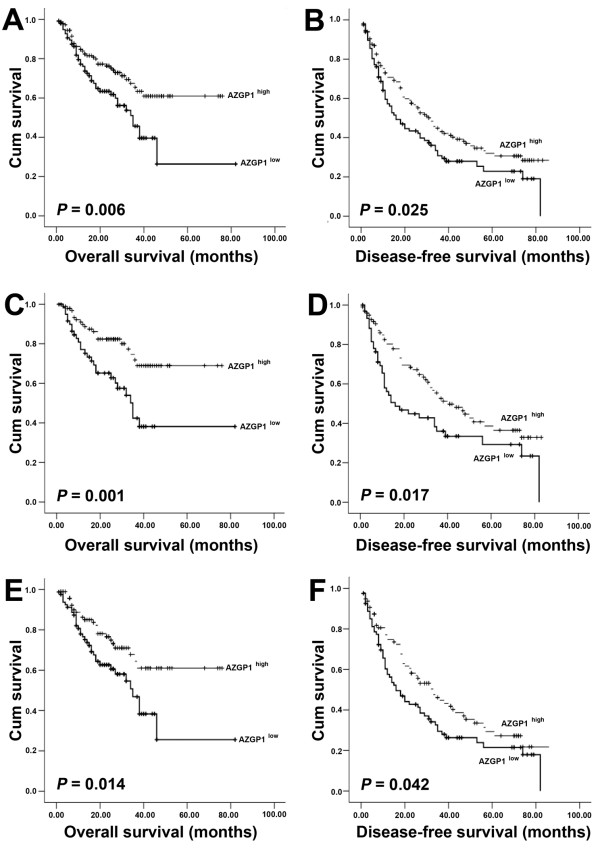
**Survival analysis of AZGP1 expression in HCC. A, B.** Probability of OS (**A**) and DFS (**B**) of total HCC patients. **C, D.** Probability of OS (**C**) and DFS (**D**) of well differentiated (grade I + II) HCC patients. **E, F.** Probability of OS (**E**) and DFS (**F**) of positive cirrhosis HCC patients.

The association between AZGP1 expression in HCC and the survival time of selected patients was analyzed with Kaplan-Meier survival analysis (Figure 3). The median overall survival (OS) time of low AZGP1 expression group was 34.1 months, significantly shorter than that of high AZGP1 expression group (72 months) (*P* = 0.006, Figure 3A). Furthermore, the median disease-free survival (DFS) time of low AZGP1 expression group was remarkably shorter than that of high AZGP1 expression group (*P* = 0.025, Figure 3B). Statistically, the 5-year OS rate and DFS rate of low AZGP1 expression group were 31.0% and 22.5%, respectively, whereas they were 44.8% and 32.0%, respectively, in high AZGP1 expression group. Moreover, we stratifiedly analyzed the subtype of patient with well tumor differentiation (OS: *P* = 0.001, Figure 3C; DFS: *P* = 0.017, Figure 3D) and liver cirrhosis (OS: *P* = 0.014, Figure 3E; DFS: *P* = 0.042, Figure 3F). Results showed that HCC patients with high AZGP1 expression survived longer.

Univariate analysis was also performed on 11 clinialpathological parameters that may affect postoperative survival of HCC patients (Table 2). Results indicated that AZGP1 expression, as well as serum AFP level, tumor size, tumor multiplicity, tumor differentiation, clinical stage and vascular invasion, was one of the factors that are responsible for efficacy of surgical treatment in HCC patient, by showing that AZGP1 expression was significantly correlated to OS (*P* = 0.007) and DFS (*P* = 0.033) of HCC patients. Next, we subjected the statistically significant parameters to Cox proportional hazards regression model to evaluate the significance of AZGP1 expression in HCC prognosis. Results suggested AZGP1 expression was not an effective independent prognostic factor (Table 3).

**Table 2:** Univariate analysis of different prognostic factors in 246 HCC patients

**Variable**	**Overall survival rate (%)**		**Disease-free survival rate (%)**	
	**HR (95% CI)**	**P value**	**HR (95% CI)**	**P value**
Age (years)	1.037(0.674-1.596)	0.867	1.120(0.816-1.539)	0.482
Gender	0.74(0.356-1.536)	0.419	1.215(0.741-1.990)	0.44
HBsAg	0.776(0.421-1.431)	0.416	1.140(0.697-1.865)	0.601
AFP (ng/ml)	3.408(2.038-5.698)	**< 0.001**	2.088(1.499-2.909)	**< 0.001**
Liver cirrhosis	1.087(0.672-1.760)	0.733	1.174(0.813-1.694)	0.392
Tumor size (cm)	2.409(1.532-3.790)	**< 10.001**	1.806(1.311-2.488)	**< 0.001**
Tumor multiplicity	3.233(2.022-5.168)	**< 0.001**	1.972(1.429-2.722)	**< 0.001**
Tumor differentiation	1.649(1.246-2.183)	**< 0.001**	1.621(1.314-2.000)	**< 0.001**
Clinical stage	3.079(2.269-4.178)	**< 0.001**	1.785(1.457-2.187)	**< 0.001**
Vascular invasion	4.704(3.027-7.311)	**< 0.001**	2.393(1.714-3.341)	**< 0.001**
AZGP1	0.553(0.359-0.851)	**0.007**	0.706(0.513-0.972)	**0.033**

*CI*, confidence interval; *HR*, hazard ratio; *AFP*, alpha-fetoprotein; *HBsAg*, hepatitis B surface antigen; *AZGP1*, zinc-alpha2-glycoprotein.

**Table 3:** Multivariate analysis of overall and disease-free survival rates of HCC patients

**Variable**	**β**	**SE**	**Hazard ratio (95%CI)**	**P value**
**Overall sruvival**				
Clinical stage	1.382	0.468	3.982 (1.590-9.974)	**0.003**
Vascular invasion	0.895	0.258	2.448 (1.477-4.059)	**0.001**
AFP	0.781	0.273	2.184 (1.279-3.730)	**0.004**
Tumor differentiation	0.599	0.299	0.550 (0.306-0.988)	**0.045**
Tumor multiplicity	0.438	0.283	1.549 (0.889-2.698)	0.122
AZGP1	-0.321	0.231	0.725 (0.461-1.141)	0.164
Tumor size	0.319	0.245	1.376 (0.852-2.224)	0.192
**Disease-free survival**				
Relapse	1.252	0.167	3.499 (2.522-4.854)	**< 0.001**
Vascular invasion	0.775	0.206	2.171 (1.449-3.253)	**< 0.001**
AFP	0.533	0.173	1.705 (1.215-2.392)	**0.002**
Tumor differentiation	0.521	0.232	0.594 (0.377-0.936)	**0.025**
Tumor size	0.361	0.18	1.435 (1.009-2.041)	**0.044**
Tumor multiplicity	0.427	0.238	1.533 (0.962-2.443)	0.072
AZGP1	-0.211	0.166	0.810 (0.585-1.121)	0.204
Clinical stage	0.188	0.286	1.207 (0.689-2.115)	0.510

*β*, Regression coefficient; *SE*, standard error; *CI*, confidence interval; *AFP*, alpha-fetoprotein; *AZGP1*, zinc-alpha2-glycoprotein.

### Association of high AZGP1 expression with long period of recurrence and distant metastasis

**Figure 4:**
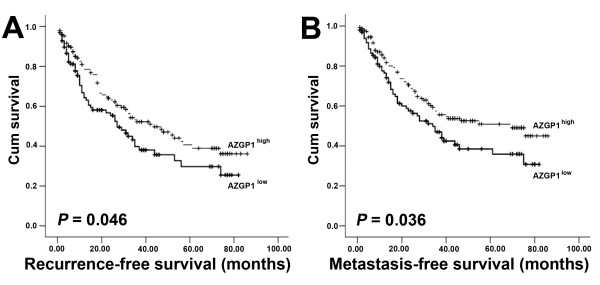
**Association of AZGP1 with HCC patients’ recurrence and metastasis. A.** Recurrence-free survival in patients stratified by AZGP1 expression. **B.** Metastasis-free survival in patients stratified by AZGP1 expression.

We further assessed whether AZGP1 expression in HCC was related to the risk of recurrence and metastasis. Survival analysis indicated that AZGP1 expression was negatively associated with the risk of clinical recurrence (HR = 0.698, 95% CI = 0.488 to 0.999, *P* = 0.046; Figure 4A) and distant metastatic progression (HR = 0.679, 95% CI = 0.471 to 0.981, *P* = 0.036; Figure 4B). This may suggest AZGP1 may be involved in tumor recurrence and metastasis.

## Discussion

Recently, it has been shown that AZGP1 plays a crucial role in carcinogenesis and is of clinicopathologic significance in human cancers [8,25-28]. In the present study, AZGP1 expression was found to be decreased in HCC tissues at both mRNA and protein levels. Moreover, low expression of AZGP1 was notably more prevalent in patients with poor tumor differentiation, serious liver cirrhosis, high serum AFP level and short survival time. Our results may suggest AZGP1 as a promising novel prognostic biomarker for HCC.

Recently reports showed AZGP1 was overexpressed in some tumors, but lost or reduced in other tumors [13,17]. Although the exact mechanism remains elusive, expression of AZGP1 is partly attributed to the acetylation status of histone which regulates gene activity by changing the conformation of chromatin. Daniel and colleagues found that AZGP1 was upregulated in lung adencarcinoma due to histone acetylation [13]. Kong et al. also suggested that AZGP1 in pancreatic ductal adenocarcinoma is lost owing to histone deacetylation [14]. However, whether histone deacetylation contributes to the decreased expression of AZGP1 found in this study requires further investigation.

Reports demonstrated that poorly differentiated tumors expressed less AZGP1 than well differentiated tumors in most of popular human tumors, such as breast cancer, prostate cancer, oral squamous cell carcinomas, epidermal tumors, sweat gland tumors, pancreatic cancer and cervical cancer [13,15,16,26-30]. Interestingly, complicated relationships between AZGP1 expression and clinical stages of cancer patients are common. AZGP1 was stage-dependent increased in the urinary bladder cancer [25]. However, an inverse association between AZGP1 expression with tumor stage was found in prostate cancer [29]. In addition, AZGP1 expression is correlated positively to leptin receptor and negatively to adiponectin receptor and estrogen receptor in breast cancer tissue. In our study, low level of AZGP1 appeared to be significantly associated with poorer differentiation. Although the percentage of patients with HBV infection was greater and the ages of patients were younger in our study, when compared to patients with HCC in Western countries or Japan, our findings suggest that loss or decrease expression of AZGP1 may promote HCC progression. Further studies should be needed to examine the role of AZGP1 in patients with HCV-related HCC or without hepatitis viral infection.

AZGP1 is considered as a potential biomarker of various tumors. AZGP1 was also proposed as predictor for breast cancer due to its elevated expression in cancer and normal epithelial adjacent tissues but not in normal tissue of healthy women [31]. AZGP1 was reported to be a diagnostic marker for cancer cachexia due to its high expression [32,33]. In prostate cancer, AZGP1 could be served as a potential serum maker, being expressed in malignant prostatic epithelium [16]. However, relationship between AZGP1 and HCC has not been studied. In the present study, we showed that AZGP1 expression was decreased by over 2 folds in HCC tissues, compared to that in the adjacent non-tumorous liver tissues. These data indicated a role for AZGP1 of a potential biomarker to give a selective advantage in the HCC tumor progression.

AZGP1 has been identified as a predictor of prognosis in some tumors. In lung adenocarcinoma and prostate cancer, patients with high levels of AZGP1 had better survival than those with low levels of AZGP1 [14,29,34]. Absent or weak AZGP1 expression is reported to be associated with shorter recurrence-free and metastasis-free survival of prostate cancer patients [18,19]. In our study, HCC patients with low AZGP1 expression had shorter survival time. Cox regression analysis indicated that AZGP1 might be one of the factors that affect HCC patients' survival. High AZGP1 expression was associated with significantly longer period of clinical recurrence and distant metastasis. These observations suggest that it might be of significance to utilize the expression of AZGP1 as a preliminary diagnostic approach to distinguish HCC from non-tumourous liver disease, and that more attention should be paid to HCC patients with decreased AZGP1 expression during and after the process of therapy. However, it is noteworthy that further investigation and validation are required before a clinical practice. Collectively, these data suggest that AZGP1 may be a promising prognostic marker for HCC.

## Conclusion

In summary, our study demonstrated that AZGP1 was frequently decreased in HCC. Low expression of AZGP1 could be served as a tumor biomarker for poor differentiation and a predictor for poor prognosis of HCC patients.

## Abbreviations

AZGP1: Zinc-alpha2-glycoprotein; HCC: Hepatocellular carcinoma; qRT-PCR: Quantitative real-time polymerase chain reaction; IHC: Immunohistochemistry; AFP: Alpha-fetoprotein; HBV: Hepatitis B virus; HCV: Hepatitis C virus; HBsAg: Hepatitis B surface antigen; MHC I: Major histocompatibility complex class I; ICGR15: The 15-min retention rate for indocyanine green; TNM: Tumor-lymph node-metastasis; TMA: Tissue microarray; OS: Overall survival; DFS: Disease-free survival.

## Competing interests

The authors declare that they have no competing interests.

## Authors’ contributions

JPY carried out and coordinated the study. YH, LZL, LLL, GBX, and YL performed the experiments; YH, LZL, CY, XZ, MYC, and Chris ZYZ analyzed the data; YH wrote the paper; All authors read and approved the final manuscript.
